# Acquired pellicle engineering: a fascinating approach to prevent demineralization

**DOI:** 10.1590/1678-7757-2024-0359

**Published:** 2025-05-02

**Authors:** Carolina Ruis FERRARI, Matthias HANNIG, Marília Afonso Rabelo BUZALAF

**Affiliations:** 1 Universidade de São Paulo Faculdade de Odontologia de Bauru Departamento de Ciências Biológicas Bauru Brasil Universidade de São Paulo, Faculdade de Odontologia de Bauru, Departamento de Ciências Biológicas, Bauru, Brasil.; 2 Saarland University Clinic of Operative Dentistry, Periodontology and Preventive Dentistry Homburg Germany Saarland University, Clinic of Operative Dentistry, Periodontology and Preventive Dentistry, Homburg, Germany.

**Keywords:** Acquired Enamel Pellicle, Cystatin, Hemoglobin, Proteins, Statherin

## Abstract

The acquired enamel pellicle (AEP) consists of an organic, acellular, and bacteria-free film, formed *in vivo* as a result of biomolecules adsorption onto the tooth surface. It is composed of proteins, glycoproteins, lipids, phospholipids, and other macromolecules, such as carbohydrates. The AEP formation process is complex and can be divided into three stages: initiation, development, and maturation. The pellicle has two main layers: the globular and basal layers. The basal layer offers the most protection against demineralization, as the subsequent globular layer is weaker and less tenacious. The formation of the AEP can be influenced by various factors, such as the physicochemical properties of the teeth, location in the oral cavity, pathologies, and even the oral microbiota. With the advancement of “omics” techniques, it has been possible to observe the presence of acid-resistant proteins in the AEP, which allowed the development of the “acquired pellicle engineering” strategy. This strategy involves enriching and modifying the basal layer with acid-resistant proteins. Among these proteins, hemoglobin, statherin-derived peptide, and a protein derived from sugarcane stand out. The objective of this literature review is to provide a comprehensive overview of the AEP, detailing its composition, formation process, and protective functions. Additionally, the review aims to explore recent advances in the field of “acquired pellicle engineering,” highlighting the acid-resistant proteins of the AEP and their potential applications in dentistry. Finally, the review intends to highlight the clinical implications of these findings and how they may contribute to the development of new strategies for the prevention and treatment of dental pathologies according to published studies.

## Introduction

The acquired pellicle was first described by Alexander Nasmyth in 1839, who stated that it was a delicate layer formed on the tooth surface.^1^ For a long time, it was believed that it was of embryonic origin.^1^ The potential discovery that the acquired pellicle may not be solely of embryonic origin came in 1926, when a study described that these dental integuments were not only present on tooth surfaces but also on dental prostheses and amalgam restorations.^2^The term “acquired enamel pellicle” (AEP) was initially introduced by Dawes in 1963 within a review regarding the nomenclature of enamel surface integuments. It refers to a protein-based film formed via adsorption onto the enamel subsequent to exposure to the oral environment.^3^ There is evidence that this pellicle can undergo remodeling due to the chemical and enzymatic factors of absorbed proteins.^4^ The AEP represents a physiological, dynamic, and constantly evolving ecological entity, demonstrating continuous adsorption and desorption of biomolecules.^5,6^It is known that the AEP consists of a thin acellular, bacteria-free organic layer, formed *in vivo* on the tooth surface as a result of the selective adsorption of proteins, originating from different fluids.^4^ Besides proteins, the AEP contains neutral lipids, phospholipids, glycolipids, and other macromolecules such as carbohydrates.^3,7,8^

The formation of the AEP occurs in three distinct stages via a selective process, involving precursor proteins such as proline-rich proteins (PRPs), statherin, cystatins, amylase, histatins, and lysozyme.^4,9,10-12^Its composition can be influenced by several individual and external factors, as well as pathological oral conditions.^13-15^ Among its layers, the basal layer is the most important, playing a critical role in the protective function of the AEP, especially in demineralization. The subsequent layers are less compact and possess lower protective ability against demineralization.^4^

**Figure 1:**
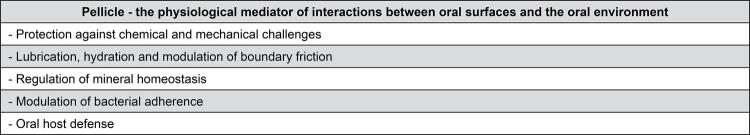
Physiological functions of the pellicle in the oral cavity

The pellicle role in shielding the tooth surface from acids is notable, as it forms a barrier capable of preventing direct contact, effectively functioning as a selective semipermeable membrane.^9^ Calcium-binding proteins, such as statherin and mucin, play a pivotal role in hindering further hydroxyapatite dissolution by adjusting calcium concentrations on the tooth surface to a supersaturated level,^16,17^thus contributing significantly to mineral homeostasis maintenance^11^ (Figure 1).

Moreover, the AEP serves as a lubricant in the oral environment, reducing friction between the tooth and adjacent structures while shielding the tooth surface from abrasion and protecting against acids.^18,19^In recent years, with the advent of proteomic studies, there has been significant progress in understanding the protein composition of the AEP,^20^ as well as the impact of the different proteins found within the basal layer, such as cystatin,^21^ statherin,^22^ and hemoglobin,^23^ which have been identified as resistant to removal by erosive and/or cariogenic acids. The identification of these proteins has led to a new approach for the prevention of oral diseases/conditions, especially demineralization, based on “acquired enamel pellicle engineering” procedures.^24,25^

In this literature review, the aim is to present the strategy of “acquired pellicle engineering” for the prevention of demineralization. We discuss key issues related to the AEP and proteins with potential acid-resistant properties, focusing on the development of preventive measures dependent on the composition, characteristics, and properties of the pellicle. This will allow a better understanding of the role of the AEP as an important biological factor with potential protective effects against demineralization and might facilitate the translation of this information into clinical practice. The action of the AEP against dental caries is still in its early stages of investigation, whereas its protective role against erosive tooth wear (ETW) has been more extensively studied and demonstrated. The potential of the AEP in modulating bacterial adhesion, altering biofilm composition, and reducing acidogenic activity is promising but remains less explored compared to its role in erosion prevention.

For this study, we searched MEDLINE (PubMed) from inception through August 2024 for articles in which the title and abstract explicitly mentioned the formation and composition of the AEP. This criterion ensures that the included literature is directly relevant to understanding the molecular composition of the pellicle and its role in protection and modification. By narrowing our selection to studies that specifically address these aspects, we aimed to provide a comprehensive review of AEP-related research based on “acquired enamel pellicle engineering.” Relevant studies were selected via independent review by two of the authors (CF and MB). Exclusion criteria were studies in which the pellicle was investigated on restorative materials, as well as studies that referred to the pellicle formed on surfaces other than enamel.

## Literature review

### Composition of the acquired enamel pellicle

**Figure 2:**
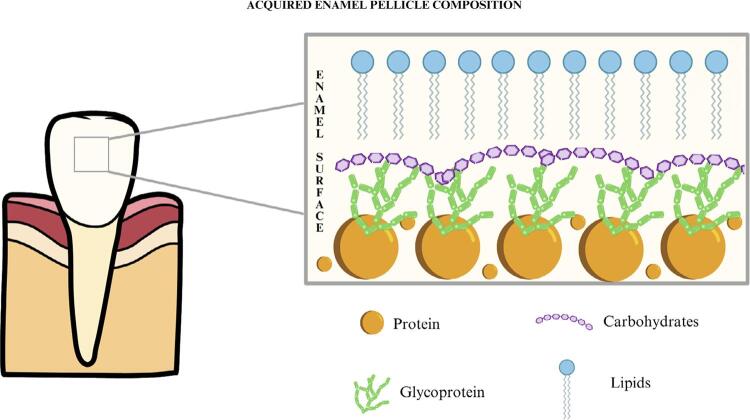
Composition of the acquired enamel pellicle

The AEP is formed by components originating from microbial products, oral mucosa, gingival crevicular fluid, and salivary gland secretion,^11,18^ predominantly composed of salivary proteins, glycoproteins, lipids, and carbohydrates (Figure 2). These organic components have a high affinity with the tooth surface and rapidly adsorb onto the enamel after tooth brushing or professional prophylaxis. The main components of the AEP are proteins and glycoproteins, as well as some amino acids, which are responsible for providing the antimicrobial effect and influencing the remineralization process.^11,26,27^ Additionally, lipids account for around 25% and ensure the permeability of the pellicle, which is responsible for being the basis of resistance to the action of acids in the oral cavity.^28-30^ Finally, carbohydrates can act as a nutrient supply for the biofilm and contribute as a protective barrier.^18^

The composition of the AEP can be influenced by the circadian rhythm, saliva composition, pathological conditions, its location in the oral cavity, and the proteolytic capacity of the oral fluid.^11,14,31,32^ The pellicle is thicker on the lingual surfaces of the lower teeth due to the presence of submandibular and sublingual glands that keep this area in constant contact with saliva.^33^ However, it has a thinner thickness on the palatal surface of upper teeth due to the friction of the tongue, which generates shear forces.^4,32,34^According to the study by Mutahar, et al.^35^ (2020), the composition of saliva from different salivary glands plays a significant role in providing protection against ETW. This is attributed to the presence of distinct proteins in the saliva produced by these glands, which contribute to variations in the protective properties of the AEP. Additionally, Baumann, et al.^36^ (2016) demonstrated that human stimulated saliva offers varying levels of protection against dental erosion compared to artificial saliva. This variation is primarily due to the protein content of human saliva, which significantly affects the formation and efficacy of the protective pellicle.

Under pathological conditions, one study indicated that the AEP in patients with gastroesophageal reflux disease and ETW exhibited a distinct proteomic profile compared to individuals without ETW.^23^ Notably, hemoglobin was found at higher levels in patients without ETW when compared to those that suffered from teeth wear, suggesting that this protein may play a protective role against dental erosion caused by intrinsic acids.^23^ An example of other neuro-hormonal influences on the salivary reflex is the circadian rhythm, which has been shown to significantly impact both the salivary flow rate, and its ionic composition.^37^ The circadian rhythm regulates physiological processes in the body over a 24-hour cycle, including saliva production.^38^ During periods of high salivary flow typically associated with daytime activity, the secretion of salivary proteins and ions is elevated, leading to a richer and more dynamic composition of saliva. Conversely, during periods of low salivary flow, such as at night, the protein concentration and ionic composition of saliva are altered.^39^ These variations in salivary flow and composition influence the formation and properties of the acquired enamel pellicle.

Another important factor that can lead to variation in the composition of the AEP is the fact that salivary gland ducts open at different locations in the oral cavity, which can even impact the protective potential of the AEP,^14^ as salivary secretion has a characteristic protein composition according to the salivary gland.^35^ The parotid gland secretes saliva characterized by proteins rich in amylase and proline, and contains small amounts of cystatins, lysozyme, and glycoprotein. In contrast, the sublingual gland secretes saliva with high concentrations of mucin and elevated levels of lysozyme. The submandibular gland contains a higher concentration of cystatin S, and finally, the palatine secretions contain high molecular weight mucins with a high concentration of amylase.^40^ Thus, considering that the AEP is mainly composed of proteins, depending on the location of the tooth in the dental arch, its thickness may vary.^14^Considering these aspects, any imbalance in the cholinergic and adrenergic systems or exogenous conditions, such as medication use, food intake, or even radiation, can produce changes in salivary flow and consequently in the composition of the pellicle.

### Proteins

Salivary proteins and glycoproteins are abundantly found in the AEP, playing fundamental roles in maintaining oral health^18^ and are being extensively studied not only in healthy contexts but also in relation to certain oral pathologies.^14, 41,42^

**Figure 3:**
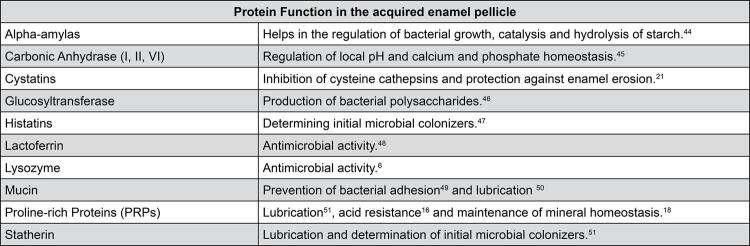
Main proteins found in the acquired enamel pellicle in *in vivo* studies

In *in vivo* studies, many proteins can be identified in the AEP, including: histatins, glucosyltransferase, carbonic anhydrase (I, II, VI), mucin, cystatin, proline-rich proteins (PRPs), and enzymes such as alpha-amylase, lysozyme, and lactoferrin. Components of the blood plasma are also present, mainly due to increased gingival fluid in cases of gingivitis. Among them are fibronectin, albumin, immunoglobulins (IgA, IgG), keratins 13 and 15, and fibrinogen.^8,15,43^Figure 3 shows the main proteins identified in the AEP in *in vivo* studies and their main functions.

Among the proteins of significant physiological relevance to the AEP, statherin provides lubrication to the oral environment, determines initial microbial colonizers,^11^ resists acid attacks,^22^ and inhibits both spontaneous calcium phosphate precipitation and crystal growth.^52^ Meanwhile, PRPs exhibit a high affinity with dental enamel, mainly due to their electrostatic interactions,^18^ besides being involved in lubrication,^11^ acid resistance,^16^ and maintenance of mineral homeostasis (maintaining a saturation state concerning calcium and phosphate in the oral cavity, inhibiting their precipitation at neutral pH).^18,21^

Another important group of proteins forming the AEP are mucins, which significantly contribute to lubrication and hydration. Additionally, they have immunological relevance^18^ and inhibit demineralization caused by erosive attacks.^53^ Histatins are considered precursors to AEP formation and are responsible for determining initial microbial colonizers,^11^ maintaining mineral homeostasis, and acid resistance.^16^

Cystatins, low molecular weight proteins, exhibit antimicrobial action^54^and acid resistance.^21^Annexin functions by binding to phospholipids of biological membranes and regulates Ca^2^ ionic currents.^55^ Carbonic anhydrase (I, II, VI) has demonstrated acid resistance^18^ and accumulates in the pellicle, maintaining its enzymatic activity. Additionally, lactoferrin, with a high molecular weight, exhibits significant antimicrobial activity.^18^ Immunoglobulins possess antimicrobial properties^54^ and are associated with the immune response.^18^ In addition to these proteins mentioned above, there are those considered typical proteins of the AEP such as Protein S100-A8, Lysozyme C, Lactoferrin, Statherin, Myeloperoxidase, and PRP 3, which were identified in proteomic studies.^14,20^

Enzymes exhibit catalytic activity, degrading salivary components and facilitating their adsorption.^56^ Considering this aspect, the most abundant enzyme is alpha-amylase, capable of hydrolyzing the alpha-1-4 glycosidic bonds of starch and glycogen polysaccharides. Moreover, it can form complexes with glucosyltransferase in the presence of amylase-binding protein, potentially influencing biofilm formation during its initial colonization phase.^56^ Another noteworthy enzyme is lysozyme, which primarily interacts with other proteins, aiding in the formation of protein aggregates in the AEP and may act as an antibacterial component.^19^

With the advancement of “omics” techniques, it has become possible to identify different types of proteins found in the AEP under various clinical conditions.^20,23,57^ Thus, the abundance of the proteins found in the AEP reflects the complexity and importance of these components in maintaining oral health. Their various functional roles can contribute to important processes such as dental de/remineralization, potentially influencing dental caries and ETW.^12,25^ Additionally, the role of these proteins in determining initial microbial colonizers can influence biofilm formation in the periodontium in cases of gingivitis and periodontitis, as well as in cases of caries.^24,58^

The AEP undergoes significant *in vivo* and proteomic compositional changes during the first two hours of its formation, a critical period for its development. Proteins such as histatin 1 and histatin 3 showed a substantial decrease in abundance after 60 and 120 minutes compared to their levels in the initial 5 minutes of *in vivo* pellicle formation.^51^ These proteins are notably abundant during the early stages of AEP development, highlighting their role as precursors in pellicle formation. In contrast, statherin maintains consistent levels throughout the formation process, suggesting that it is less prone to proteolytic degradation than histatins. Furthermore, salivary proteins, such as amylase, lysozyme, and lactoperoxidase, demonstrated a progressive increase in abundance during *in vivo* AEP development, which may be attributed to their protein-protein interaction capabilities that support their integration into the pellicle and their contributions to its maturation.^51^

### Lipids

Lipids are typically derived from the major salivary glands^59^ and represent approximately 25% of the composition of the AEP.^28^Lipid components have a significant impact on AEP formation, especially on adsorbed molecules, its ultrastructure, and density.^60^ The earliest attempts to investigate lipid components in pellicle samples were conducted by Slomiany, et al.^61^ (1986). This study identified free fatty acids, cholesterol, cholesterol esters, triacylglycerols, glycolipids, and phospholipids as constituents of the AEP layer, suggesting a correlation between their proportions and the resistance of the film to acids.^61^

Upon evaluating the lipidome of the AEP *in situ*, it is consistently possible to detect fatty acids; during the first 2 hours of pellicle formation, an increase in fatty acid content can be observed.^62^ Additionally, the intraoral location may not influence the composition of this element in the pellicle, since the samples of pellicles formed bucally and palatally have an almost identical profile after different exposure times.^62^However, it should be highlighted that while the proteome of the AEP has been extensively investigated under different circumstances, only a few studies explored the lipid composition of this film. Regarding the presence of phospholipids, four types were identified: lysophosphatidylcholine, phosphatidylcholine, phosphatidylethanolamine, and phosphatidylinositol.^62^Finally, triglycerides were identified, and it is relevant that neither monoacylglycerols nor diacylglycerols were identified in any of the samples investigated in the study performed by Reich, et al.^62^ (2022). Thus, it can be stated that a characteristic profile of fatty acids, as well as specific phospholipids and triglycerides, was detected.^62^

In summary, lipids can interact with other components to contribute to the permeability of the AEP, influence its ultrastructure, and affect the initial stage of bacterial adhesion to dental surfaces, primarily due to their hydrophobic nature.^29^

### Carbohydrates

The AEP presents carbohydrates among its components. They are mostly found in the form of complex compounds such as glycoproteins and glycolipids.^13^ Glucose in particular can be derived from both salivary glycoproteins and bacterial glucans.^13^ Although its specific function in the AEP still lacks in-depth analysis,^63^ preliminary studies suggest that these carbohydrates may serve as a nutrient source for the biofilm.^13,18^

### Acquired pellicle formation

**Figure 4:**
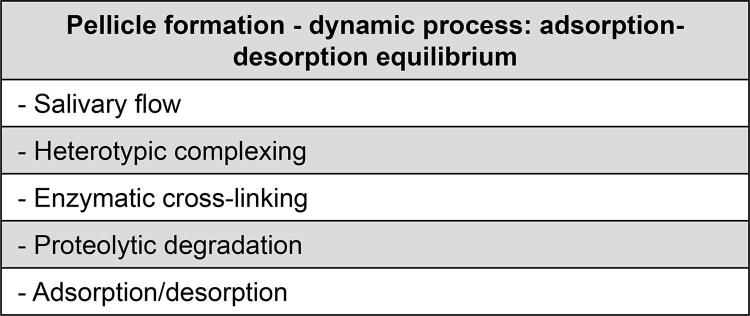
Processes involved in pellicle formation and adsorption-desorption equilibrium

**Figure 5:**
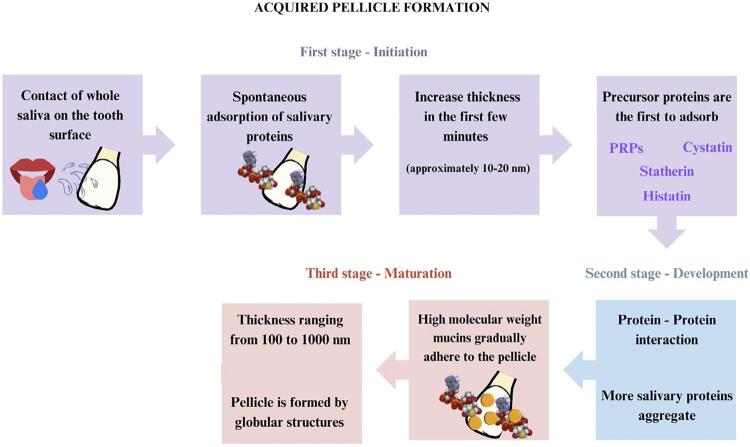
Acquired pellicle formation

The process of AEP formation is selective, dynamic, and can be influenced by adsorption and desorption processes^13^ (Figure 4). It is divided into different stages: initiation, development, and maturation (Figure 5).^9^In the first stage, AEP formation starts by spontaneous adsorption of salivary proteins onto the tooth surface.^4,32^ There is an increase in its thickness (approximately 10**–**20 nm) in the first few minutes, remaining stable for 30**–**45 minutes depending on the location in the oral cavity.^4,32^

The enamel surface has a predominantly negative charge conferred by the phosphate ions. This negatively charged surface is then coated with a layer of calcium ions. In this way, salivary proteins are adsorbed to the enamel due to electrostatic interactions with the calcium and phosphate ionic layer.^64^

Precursor proteins are the first to adsorb due to their strong binding affinity to hydroxyapatite, interacting with tooth enamel, which renders the AEP negatively charged, characterized as an electron-dense layer,^10^ known as the basal layer.^4^ Among these proteins are PRPs, statherin, cystatins, amylase, histatins, and lysozyme.^11^ They can interact with calcium and phosphate ions on the enamel surface via ionic, hydrophobic, and van der Waals interactions.

The basal layer of the AEP plays an extremely important role in its protective capacity since the subsequent layers are less dense.^4^ It has been reported that there is no difference in the protective potential against demineralization of pellicles formed after 3 minutes of exposure to saliva, compared to those formed after 2 hours,^56^ highlighting the protective role of precursor proteins. Therefore, the basal layer of the AEP is the most important for protection of the tooth surface, mainly because it is not completely removed during erosive challenges.^19^

In the second stage, known as development, more salivary proteins aggregate with the precursor proteins of the pellicle, resulting in protein-protein interactions on the tooth surface, which aggregate into a globular form.^19^ The diameter of the built globular structure and the thickness of the salivary film continue to increase until reaching maturation within hours.^13^

Finally, in the maturation stage, high molecular weight mucins gradually adhere to the pellicle,^65^ and there is a rapid increase in its thickness (100**–**1000 nm), which, together with the presence of globular structures, suggests the involvement of protein aggregates, rather than individual proteins, in its development.^4,66^ The AEP thickness reaches a plateau between 30 and 90 minutes, with a thickness ranging from 100 to 1000 nm, depending on its location in the oral cavity, being thicker in the vestibular region when compared to the lingual.^4^

The AEP can undergo intrinsic and extrinsic maturation, which can affect its solubility.^11^ Intrinsic maturation can be caused by the presence of transglutaminase, derived from oral epithelial cells, which can crosslink between basic PRPs and statherin,^8,67^ as well as by the presence of alkaline phosphatase. Indeed, enzymatic crosslinking and dephosphorylation appear to be the most important events for the intrinsic maturation of the pellicle, while proteolysis seems to be of lesser importance.^67^ On the other hand, salivary proteolysis, which can occur before or after adsorption to hydroxyapatite, plays an important role in the extrinsic maturation of the pellicle, as many of its components are peptide fragments. Furthermore, the formation and maturation of the AEP can be influenced by external factors like dental products (toothpaste and mouthwash solutions) and the individual’s diet.^68^ Even pathological oral conditions, such as gingivitis, can alter the proteolytic capacity of saliva, causing changes in the properties of salivary proteins due to the increased flow of crevicular fluid and plasma proteins,^15^ which changes the composition of the AEP.

### Acquired enamel pellicle engineering

The AEP plays a crucial role in protecting against dental demineralization and regulating the oral microenvironment.^9^ This is due to the formation of the first layer of the AEP, in which precursor proteins with strong affinity for hydroxyapatite develop electrostatic interactions with the enamel, being absorbed onto this surface.^10^ This first layer is called the basal layer, which is characterized by providing greater protection against dental demineralization due to its electron density.^19^ This ensures that its components are not (completely) removed from the enamel surface even after severe erosive challenges.^19^ In this context, the investigation of proteins resistant to removal by erosive acids is of great importance, aiming at therapeutic strategies for dental erosion, which is characterized as the main factor for the development of ETW.^69^

The first study using the context of enriching the pellicle via pre-treatment before its formation opened a new path for the prevention of ETW.^70^ Subsequently, treatments with mucin, casein, and a mixture of these proteins was used to demonstrate the impact of modifying the AEP and its influence on the initial adhesion of colonizing bacteria. As a result, these treatments were able to decrease the number of bacteria adherents to the enamel surface.^71^ Additionally, other studies were conducted aiming to modify this organic layer, using different treatments: milk,^72^ polyphenols,^73,74^ or combinations of polyphenols and lysozyme.^75^

The advancement of “omics” techniques, especially proteomics, significantly contributes to deepening the knowledge of the AEP composition.^11^ This is due to the shift from previously employed enzymatic or immunological techniques to a high-sensitivity approach of proteomic techniques, enabling the identification of many proteins simultaneously.^11^ With this revolutionary technique, potential biomarkers for oral health conditions can be identified.

The proteomic analysis of the AEP has been essential in elucidating the complex function and interaction between the proteins present in this pellicle and the dental surface.^20^ Proteomics offer a systematic and comprehensive approach for identifying and quantifying the proteins present in the AEP, as well as their post-translational modifications and protein-protein interactions. This technique enables a detailed analysis of the molecular characteristics of the pellicle, providing valuable insights into its biological function, not only in terms of health but also in relation to certain pathologies.

Analyzing the proteomic profile of the AEP enables the identification not only of abundant proteins but also of low-abundance ones. This is crucial considering that many proteins with important roles in the pellicle may be expressed at low levels, especially when comparing healthy individuals with those that suffer from certain pathologies, which provides insights into the underlying mechanisms of pathological conditions and enables the development of new diagnostic and treatment strategies.

**Figure 6:**
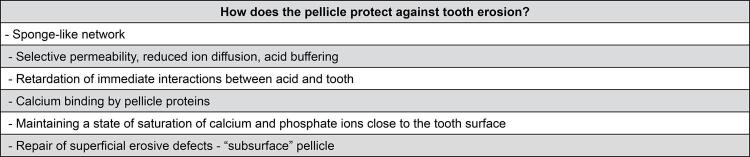
Aspects of pellicle protection against dental erosion

In this context, the incorporation of proteins into the AEP can affect its ability to protect against ETW (Figure 6). Patients with ETW have half the amount of proteins in the *in situ* formed pellicle compared to the control group, reinforcing the protective role of the proteins present in the AEP.^76^ In addition, exposure to acids removes the outer globular layer of the pellicle, leaving the basal layer intact.^18^ Therefore, it is crucial to identify which proteins present in the AEP are capable of conferring protection against enamel dissolution by acids.

Using proteomics, proteins resistant to removal by extrinsic acids (citric acid) were identified, among which mucins and cystatins stood out.^21,77^ Among them, cystatins seem to be quite promising, as the relative expression of cystatin B was found to be increased in the AEP 20 times after pellicle exposure to citric acids and 13 times after exposure to lactic acids, simulating erosive and cariogenic challenges.^21^ Proteins resistant to removal by intrinsic acids were also identified.^22^ This topic is extremely important, as the pH of gastric acids is lower and their buffering capacity is higher than that of dietary acids, usually leading to more severe dental tissue destruction.^78^ In this sense, it was observed that statherin remained in the *in vivo* AEP after challenges with 0.01 M HCl (pH=2) and 0.1 M HCl (pH=1), even in cases in which the AEP formed in the short-term (for only 3 min).^22^

An important condition associated with intrinsic erosion, gastroesophageal reflux disease (GERD), affects about 10 to 20% of the population, increasing the need to develop new preventive and therapeutic measures. In a study, the protein profile of the AEP, collected from the vestibular surface, was evaluated in volunteers with GERD without ETW and compared to those with GERD and dental erosion as well as to control patients (without GERD and ETW).^23^ The main objective was to find AEP proteins capable of explaining the greater resistance to ETW in volunteers with GERD and without ETW compared to others. In total, 458 proteins were identified, with 76 common to all three groups.^23^ In the quantitative analysis, when the group of GERD patients without ETW was compared to the group of GERD patients with ETW, the proteins that caused the greatest reduction in expression were Lysozyme C, Antileukoproteinase, Cathepsin G, neutrophil defensins, and basic proline-rich proteins (PRPs), while those that caused the greatest increase in expression in the first group compared to the second were several hemoglobin subunits, followed by albumin and cystatin isoforms.^23^Among these proteins with differential expression, hemoglobin stands out, as several subunits were expressed more than three times higher in the group of GERD patients without ETW compared to those with the same disease but presenting erosive lesions.^23^

In relation to dental caries, it was shown that cystatin B was increased by more than 13 times in the AEP after exposure to lactic acid,^77^ suggesting protection conferred by the acquired pellicle against acids from cariogenic challenges. Besides the ability of the AEP to reduce the diffusion of acids to the tooth surface, it is known that the molecular and physicochemical composition of the pellicle is fundamental in determining the pattern of microbial colonization.^79^ The intimate interaction between AEP and bacteria is extremely important to understand how the biofilm develops and evolves over time, considering that the AEP has sites for bacterial adhesion, significantly implicating in the initial colonization of dental biofilm.^79^ Thus, initial colonizers, such as *Actinomyces* spp., S. *oralis*, S. *mitis*, and S. *gordonii* influence the formation and composition of the mature biofilm,^80^ as they provide optimal conditions and substrates for the adhesion of secondary colonizers, such as S. *mutans, Fusobacterium nucleatum*, and *Veillonella* spp. Thus modifying the protein profile of the AEP by incorporating certain proteins that interfere with the initial bacterial colonization, altering the entire structure of the biofilm.

Considering these procedures, the concept of “acquired pellicle engineering” was proposed.^25^ One of its main approaches is the modification of the composition of the AEP basal layer, avoiding dental demineralization by non-bacterial acids (causing dental erosion) and bacterial acids (causing dental caries). Although advances in “acquired pellicle engineering” are promising, there are still challenges to overcome. The complexity of the interaction between salivary, bacterial, and dental enamel components demands a deeper understanding of the mechanisms involved in the formation and maintenance of the AEP.

### Sugarcane derived protein (CaneCPI-5)

Research has demonstrated a 20- and 13-fold increase in cystatin B levels within the AEP when subjected to citric and lactic acid challenges *in vivo*, simulating erosive and cariogenic conditions, respectively.^21^ This indicates that this protein might be a favorable candidate for incorporation into dental products aimed at protecting against ETW and dental caries. Nonetheless, the high cost of human recombinant cystatin makes its inclusion in dental products difficult. Recognizing the economic and technological importance of its integration into dental formulations, a recombinant cystatin sourced from sugarcane named CaneCPI-5 was developed.^81^ This protein has several advantageous properties. It demonstrates great solubility when produced via bacterial expression systems (*E. coli*), rendering its purification financially viable.^82^ Additionally, it exhibits inhibitory activity against cysteine peptidases (cathepsins B and L),^83^ which inhibit cysteine cathepsins. Furthermore, it possesses strong binding affinity to hydroxyapatite, a property that allows the protein to adsorb to dental enamel or modify the AEP, providing additional protection to the underlying enamel^84^ by reinforcing the basal layer of the pellicle. Additionally, CaneCPI-5 also increases the electron-donor sites on the enamel surface. This favors the adsorption of cationic species such as Ca^2^ and Ca(H_2_PO_4_) (calcium phosphate monobasic) as well as cationic acid-resistant salivary proteins.^85^

The first study conducted to demonstrate the promising potential of CaneCPI-5 in enamel erosion *in vitro* was performed by Santiago, et al.^81^ (2017). The authors showed that this protein is indeed capable of modifying the AEP, enhancing its protective effect against demineralization.^81^ Follow-up studies have been carried out using various *in vitro*^84^ and *in vivo*^86^ methodologies, including erosion associated with enamel and dentin abrasion *in situ*.^87^ Moreover, *in vivo* studies have demonstrated that rinsing with a solution containing 0.1 mg/ml CaneCPI-5 for one minute resulted in an increase of acid-resistant proteins within the formed AEP, both in the short-^12^ and long-term.^25^

Several studies have evaluated the combination of this protein with other components to further enhance its effect. The first study to assess the association of CaneCPI-5 with other components was conducted by Pelá, et al.^88^ (2022), in which it was observed that the combination with fluoride was the most effective treatment when compared to all other evaluated treatments.^88^ Another combination evaluated was that of CaneCPI-5 and vitamin E to prevent dental erosion, which resulted in a synergistic effect of the two components, with their association providing more protection than commercial mouthwash containing stannous and fluoride.^89^

In addition to its protective effect against the action of extrinsic acids, CaneCPI-5 has demonstrated efficacy in preventing dental caries in an *in vitro* study model via “acquired pellicle engineering” procedures.^24^ Its effect in reducing carious demineralization may be attributed to the modulation of initial colonizers in the dental biofilm, driven by alterations in the composition of the AEP. In turn, these changes influenced bacterial species, as evidenced by a reduction in acidogenic and aciduric species such as *Lactobacillus* sp., total streptococci, and *Streptococcus mutans*.^24^ This shift resulted in a significant decrease in mineral loss and lesion depth, as demonstrated using a microcosm biofilm model.

Furthermore, apart from its abilities to mitigate erosion and reduce dental caries, CaneCPI-5 also exhibits anti-inflammatory characteristics.^90^ Research has demonstrated that this cystatin can mitigate inflammation induced by subcutaneous sponge implants in mice, thereby promoting angiogenesis and fibrogenesis, crucial processes for tissue repair.^90^ CaneCPI-5 has the capacity to reduce the levels of pro-inflammatory cytokines, such as TNF-α, as cathepsin B is involved in the post-translational modification of TNF-α and regulates the trafficking of vesicles containing this cytokine.^90,91^ Moreover, it shows potential in enhancing vascularization in a dose-dependent manner, leading to increased levels of pro-angiogenic cytokines such as vascular endothelial growth factor (VEGF) and fibroblast growth factor (FGF).^90^ CaneCPI-5 exhibits this effect because, during the inflammatory response, cathepsins B and L are associated with the activation of the complex responsible for the release of cytokines (such as IL-1B and IL-18), which bind to the cell membrane and activate signaling pathways.^92^ However, CaneCPI-5 has the ability to inhibit cathepsin B, which is involved in the post-translational processing of TNF-alpha. The inhibition of cathepsins B and L may have a pro-angiogenic effect.^90^These findings point to a potential preventive effect of CaneCPI-5 against gingivitis and periodontitis, which deserves further investigation.

Despite possessing these beneficial properties, CaneCPI-5, even at high concentrations, does not exhibit cytotoxic effects on human gingival fibroblasts,^93^ making it an excellent candidate for inclusion in oral hygiene products.

### Hemoglobin

Hemoglobin (Hb) is not traditionally included in the protein components of the AEP. This protein was identified in the last few years exclusively in the posterior region of the dental arches.^14^ This may have been the reason why it had not been identified in the AEP in previous proteomic studies, as they collected the pellicle only from the anterior teeth.^27,42^

The affinity of Hb for hydroxyapatite is already known, as hydroxyapatite columns exhibit excellent performance for hemoglobin purification.^94^ Interestingly, the adsorption of hemoglobin to hydroxyapatite increases as the pH decreases, which can be explained by the electrostatic interactions between Hb molecules and hydroxyapatite, occurring through van der Waals forces, electrostatic interactions, or hydrophobic interactions. The isoelectric point of Hb is around 6.8-7.0, which means that this protein becomes positively charged when the pH is below 6.8.^95^

Interestingly, a study reported that Hb levels were 2**–**3 times higher in the AEP^23^and more than 22 times in saliva^96^ in patients with GERD without ETW, compared to patients with GERD with ETW. An *in vitro* study found that Hb at a concentration of 1.0 mg/ml was able to protect enamel against initial erosion, which indicates that this protein could be a candidate for inclusion in dental prophylactic products for ETW protection.^96^ Although the exact mechanisms of Hb interaction in the AEP remain largely unknown, its potential as a protector against intrinsic acids has been reported.^96^ *In vivo* studies have demonstrated that Hb increases protective proteins of the initial AEP, significantly altering its basal layer, and enhancing its protective function.^12^

Additionally, as this protein alters the basal layer of the AEP, consequently, it can change the composition of the subsequent layers, influencing the microbial population of the biofilm.^97^ Hemoglobin can alter the adhesion of bacteria associated with a cariogenic biofilm, exhibiting an antimicrobial effect.^97^ It was shown that Hb promoted a reduction of biofilm viability to the same extent (50%) as that provided by chlorhexidine.^97^ It is worth noting that most of the biological processes affected when comparing the Hb solution with the negative control group are relevant for the Hb anticaries activity, such as antimicrobial defense, humoral immune response and humoral antimicrobial immune response mediated by antimicrobial peptides.^98^ Since it has abundant serine, threonine, and tyrosine residues that are prone to phosphorylation, this can make the protein negatively charged, which may increase its ability to bind to calcium ions in hydroxyapatite.^99^

### Statherin-derived peptide (StatpSpS)

The search for proteins that are resistant to removal by intrinsic acids^22^ is of utmost importance, given that the gastric acids pH is lower, and their buffering capacity is higher than that of dietary acids, leading to more severe dental tissue destruction.^78^ Additionally, it has been reported that patients with eating disorders are at a higher risk of erosion (OR = 12.4), which further increases when self-induced vomiting occurs (OR = 19.6).^100^ It was observed that statherin remained in the AEP *in vivo* after challenges with HCl 0.01 M (pH = 2) and HCl 0.1 M (pH = 1), even in cases of AEP formed in a short period (only 3 minutes).^22^ Statherin is a phosphorylated salivary protein with 43 amino acid residues; its primary sequence is similar to osteopontin and casein and it has calcium-binding capacity. Its density of negative charges (due to phosphorylation of serines 2 and 3) and helical conformation in the N-terminal region are important for interaction with hydroxyapatite,^101^ as confirmed in experiments involving solid-state nuclear magnetic resonance.^102^

Additionally, an *in vitro* study reported that at least 15 N-terminal residues or more in peptides derived from statherin are required for a reduction in enamel demineralization.^103^ Computational analysis confirmed that the phosphorylation of serines 2 and 3 and the helical conformation in the N-terminal region of statherin, specifically residues 1**–**15, contain the majority of the binding energy with hydroxyapatite.^104^ These data indicate that statherin is an AEP protein resistant to removal by intrinsic acids and, therefore, has great potential to be incorporated into dental products such as mouth rinses for ETW prevention. Epidemiological data support these findings, as in patients with dental erosion, the concentration of statherin in the AEP is reduced by 35%.^76^ Reduction in statherin concentration was also observed in AEP collected *in vivo* from regions with erosion compared to regions without erosion in the same patient.^105^ Thus, peptides derived from statherin with at least 15 N-terminal residues appear to be excellent candidates for protecting against ETW when adsorbed to the enamel surface.^105^

Considering that at least 15 N-terminal residues are required to protect against ETW, an *in vitro* study demonstrated that a peptide derived from statherin, with 15 N-terminal residues, with serines 2 and 3 phosphorylated (StatpSpS) at a concentration of 1.88x10^-5^M, has potential protective effects against intrinsic initial erosion.^106^ StatpSpS, in the form of a mouth rinse, yielded promising results, as rinsing with the peptide before the formation of AEP allowed protection against demineralization from subsequent erosive challenge with citric acid *in vivo*.^25^ Additionally, in the AEP formed in 3 minutes after rinsing with StatpSpS the basal layer of the AEP is formed with large amounts of this peptide due to the high affinity of StatpSpS for hydroxyapatite, leaving less space for other proteins to adsorb.^107^ In the long-term pellicle (formed in 2 hours), the proteome becomes much richer, but several proteins decreased in the short-term remain decreased in the long-term, most of them being typical pellicle proteins. Additionally, some acid-resistant proteins, such as hemoglobin, are found exclusively in the treated group compared to the control.^107^ Besides yielding positive results in mouth rinses, StatpSpS also demonstrated potential in protecting against intrinsic dental erosion in other application vehicles, such as gel and solutions, especially in prolonged challenges.^108,109^

### Future perspectives of “acquired pellicle engineering”

The concept of “acquired enamel pellicle engineering” introduces innovative opportunities for advancing preventive and therapeutic strategies in oral health. This approach leverages the protective role of AEP and enhances its functionality by incorporating bioactive molecules. In the context of ETW and dental caries, targeted modifications of the AEP could revolutionize current treatments for enamel protection and inhibit early damage. Individuals primarily exposed to extrinsic acids may benefit the most from CaneCPI-5, while those with gastroesophageal reflux or bulimia (intrinsic acids) may benefit more from treatments containing hemoglobin or statherin. The incorporation of these proteins into formulations such as mouthwashes and dentifrices represent a promising step forward.

Manipulating the composition of the AEP offers a promising strategy for influencing the adherence of periodonto-pathogenic bacteria, thereby supporting the control and management of the oral microbiota. This can play a crucial role in reducing the inflammation and tissue destruction associated with periodontitis. By selectively enhancing the protective proteins present in the AEP, it may be possible to create an environment that discourages the colonization and proliferation of pathogenic bacteria, while promoting the stability of a healthy microbial community.

The development of novel biotechnological approaches has expanded to include laboratory modifications of AEP. Proteins such as statherin, histatins, and proline-rich proteins, which already possess inherent antimicrobial or protective properties, can be optimized or hybridized to enhance their functionality. This hybridization could involve combining the beneficial attributes of multiple proteins to create engineered molecules with superior efficacy in bacterial inhibition, biofilm control, and enamel protection. While these strategies are highly promising, the field of protein hybridization for AEP enhancement is still in its infancy. Significant research is required to: 1) elucidate protein-protein interactions: understanding how AEP proteins interact with one another, with hydroxyapatite, and with bacterial surfaces is critical for designing effective hybrids; 2) optimize stability and safety: engineered proteins must be stable in the oral environment and safe for long-term use, without unintended effects on the oral microbiome or systemic health; 3) evaluate *in vivo* efficacy*:* laboratory findings need to be validated in clinical studies to determine their real-world effectiveness in managing periodontitis and maintaining oral health.

The integration of AEP recombinant hybrid proteins into oral care products represents an exciting frontier in biotechnology. Mouthwashes, dentifrices, gels, or slow-release formulations containing these engineered proteins could provide a targeted approach to managing periodontitis, ETW and dental caries. Finally, while the concept of AEP protein hybridization holds immense potential, a multidisciplinary effort involving microbiology, biochemistry, and clinical research is essential to translate these innovative ideas into practical and effective solutions for oral healthcare.

## Conclusion

Understanding the formation and composition of the AEP is crucial for the application of the “acquired enamel pellicle engineering” approach in protecting the tooth surface. The basal layer of the pellicle can be enriched with acid-resistant proteins, such as CaneCPI-5, hemoglobin, and StatpSpS, improving its ability to protect the teeth against acids from both non-bacterial and bacterial origin. Using this engineering, new therapeutic approaches and dental products can be developed, enhancing oral health and contributing to the prevention of pathological conditions. While CaneCPI-5, Hemoglobin, and Statherin each hold potential for modifying the AEP and enhancing enamel protection, their limitations—ranging from staining and degradation to interactions with other molecules—must be carefully considered. More research is essential to optimize their use, evaluate their long-term effects, and ensure their safety and efficacy in clinical applications.

Since the AEP is predominantly composed of proteins, its protein-protein interaction mechanisms can be further explored via proteomic techniques, providing valuable insights. These techniques allow for a deeper understanding of the mechanisms involved in protein adsorption on the tooth surface, contributing to the development of more effective dental protection strategies.
